# A Core MYC Gene Expression Signature Is Prominent in Basal-Like Breast Cancer but Only Partially Overlaps the Core Serum Response

**DOI:** 10.1371/journal.pone.0006693

**Published:** 2009-08-19

**Authors:** Sanjay Chandriani, Eirik Frengen, Victoria H. Cowling, Sarah A. Pendergrass, Charles M. Perou, Michael L. Whitfield, Michael D. Cole

**Affiliations:** 1 Department of Molecular Biology, Princeton University, Princeton, New Jersey, United States of America; 2 Department of Pharmacology and Toxicology, Dartmouth Medical School, Lebanon, New Hampshire, United States of America; 3 Department of Genetics, Dartmouth Medical School, Lebanon, New Hampshire, United States of America; 4 Department of Genetics and Pathology, Lineberger Comprehensive Cancer Center, University of North Carolina at Chapel Hill, Chapel Hill, North Carolina, United States of America; 5 Department of Medical Genetics, Ullevål University Hospital and Faculty of Medicine, University of Oslo, Oslo, Norway; National Institute on Aging (NIA), National Institutes of Health (NIH), United States of America

## Abstract

**Background:**

The MYC oncogene contributes to induction and growth of many cancers but the full spectrum of the MYC transcriptional response remains unclear.

**Methodology/Principal Findings:**

Using microarrays, we conducted a detailed kinetic study of genes that respond to MYCN or MYCNΔMBII induction in primary human fibroblasts. In parallel, we determined the response to steady state overexpression of MYCN and MYCNΔMBII in the same cell type. An overlapping set of 398 genes from the two protocols was designated a ‘Core MYC Signature’ and used for further analysis. Comparison of the Core MYC Signature to a published study of the genes induced by serum stimulation revealed that only 7.4% of the Core MYC Signature genes are in the Core Serum Response and display similar expression changes to both MYC and serum. Furthermore, more than 50% of the Core MYC Signature genes were not influenced by serum stimulation. In contrast, comparison to a panel of breast cancers revealed a strong concordance in gene expression between the Core MYC Signature and the basal-like breast tumor subtype, which is a subtype with poor prognosis. This concordance was supported by the higher average level of MYC expression in the same tumor samples.

**Conclusions/Significance:**

The Core MYC Signature has clinical relevance as this profile can be used to deduce an underlying genetic program that is likely to contribute to a clinical phenotype. Therefore, the presence of the Core MYC Signature may predict clinical responsiveness to therapeutics that are designed to disrupt MYC-mediated phenotypes.

## Introduction

The MYC proto-oncogene is an essential gene whose function is required for normal mouse and fly development [1]–[3]. MYC is a transcription factor that can both positively and negatively regulate target gene expression (review [4]). Enormous strides have been made in understanding the biochemical properties of the MYC protein. Several nuclear cofactors such as TRRAP, RUVBL1/Tip49, p300 and SKP2 have been shown to be required for MYC induced transformation or transactivation [5]–[9]. A large list of MYC target genes forms the core of a publicly available MYC database [10]. Similarly, exhaustive studies have recognized MYC's ability to increase cellular proliferation, transformation, apoptosis, and genetic instability, as well as to inhibit cellular differentiation (reviewed in references [11]–[13]). Despite these advances, the extraordinarily complex cellular responses to MYC expression has made it difficult to decipher the target genes that mediate these biological activities.

When translocated, amplified or misregulated, MYC can function as a potent oncogene, and it is estimated that 20% of all human cancers harbor an oncogenic allele of MYC (review [14]). MYC deregulation has been directly implicated in many specific types of cancer, including breast cancer. An early example of aberrant MYC expression in breast tumor tissue described gene amplification in 32% of 121 breast tumors [15]. A comprehensive review finds a consistent link between breast cancer and MYC amplification or overexpression. These and other data linking MYC deregulation with malignant transformation unequivocally reveal MYC's oncogenic potency, however the underlying transcriptional mechanisms driving transformation remain unresolved.

MYC overexpressing cells exhibit reduced dependence on serum for rapid proliferation and survival in culture [16], [17]. It is commonly believed that MYC enables cells to develop such independence by directly or indirectly inducing or repressing those genes that are normally regulated by serum stimulation. However, there has never been a systematic analysis of this question. In vivo, most cells experience serum stimulation normally only in the context of local injury. The gene expression program in fibroblasts in response to serum exhibits many features of the wound healing response [18]. Motivated by the observation that wound healing and tumor growth share many common physiological characteristics (review, [19]), Chang and colleagues showed that the expression of the serum response in cancer cells is a predicator of cancer patient survival and metastasis [20]. More recently, they propose a model in which the wound signature in breast cancer is induced by the coordinate amplification of MYC and CSN5, a component of a ubiquitin ligase complex [21].

New technologies are beginning to permit a better appreciation of the complexity of the cellular response to MYC. Numerous high-throughput studies querying MYC regulated genes have recently been published [22]–[32]. While many studies have focused on the discovery of MYC target genes, few have examined the complete cellular transcriptional changes induced by MYC. Interestingly, different studies identify very different MYC responsive genes. Many experimental variables like cell type, MYC protein levels, length of time of aberrant MYC expression, etc. are likely to account for the study-to-study discrepancies. In this study, we have attempted to define a core MYC-responsive gene expression signature that is less subject to many of the common experimental variables. We identified a gene expression signature common to two cell culture models of MYC overexpression and compare this profile to the gene expression data from the serum response and from primary human breast tumors. This approach allowed us to isolate the transcriptional response to MYC in a controlled system and to then show that the core MYC signature is present in a gene expression data set of breast cancer samples, which identifies the basal subtype of breast cancer and may indicate one of the genetic determinants of this group.

## Materials and Methods

### Cell Culture, Transfection and Retroviral Infection

MYCN-ER^TM^ was created as a fusion between mouse MYCN C-terminus and a portion of the estrogen receptor [33]. Stably expressing oligoclonal cultures of primary human foreskin fibroblasts were created using the LPCX vector. The constitutive MYCN expression used a Flag-tagged mouse MYCN cDNA in the LXSH retroviral vector. MYCNΔMBII has been previously described [34]. Primary human foreskin fibroblasts (BJ, ATCC product #CRL-2522) and retroviral producer PhoeNX cells (ATCC product #SD 3443) were cultured in DMEM supplemented with 10% fetal bovine serum. Retroviral infection of BJ cells was performed using PhoeNX cells. To obtain a transduced population, retrovirus infected cells were selected for resistance to puromycin (0.8 µg/ml) or hygromycin (150 µg/ml) for 7 days.

### Western blotting

Protein lysates were resolved by standard SDS-PAGE methods. Proteins were transferred to PVDF and membranes were then blocked for 10′ to O/N in TBS (+0.1% Tween 20 and 1% skim milk). Anti-FLAG western blots were probed with diluted (1∶5000) FLAG antibody (M2, Sigma) in blocking solution. Blots were washed 3 times with TBS (+0.1% Tween 20). Diluted (1∶10,000) secondary antibody (HRP conjugated goat anti-mouse) was used to detect primary FLAG antibody. Enhanced chemoluminescence reagents (Amersham Biosciences) were used to detect HRP after exposure to Kodak XAR film.

### RNA preparation, labeling, and microarray hybridization

Total RNA was prepared from cells at times and conditions indicated in text using the TRIzol reagent (GIBCO-BRL) according to the manufacturer's protocol. Reference RNAs were made from rapidly dividing untransduced BJ cells. The Agilent low input fluorescent linear amplification kit was used to generate labeled cRNA for time course experiments from 0.5 µg of total input RNA. Cy3-CTP and Cy-5 CTP were obtained from Perkin Elmer/NEN Life Sciences. The Agilent fluorescent direct label kit was used to generate labeled cDNA for steady state experiments from 10 µg of total input RNA. A total RNA reference sample was prepared from logarithmically growing normal primary BJ fibroblasts. Reference RNAs were prepared separately for the time course experiments and the steady state experiments. Experimental samples labeled with Cy5 and reference samples labeled with Cy3 were competitively hybridized to Agilent human 1A version 2, 22,000 element arrays in both the time course and steady state studies. The exceptions were the five vector control arrays in the time course study, which were hybridized to Agilent human 1A version 1 arrays.

Hybridizations and washes were performed according to the manufacturer's protocol. Washed arrays were scanned using the Axon Instruments GenePix 4000B microarray scanner and intensities collected by using GenePix5.0 software. All reported microarray data are MIAME compliant and are stored in the UNC Microarray Database and the Gene Expression Omnibus (GEO). The data can be accessed at https://genome.unc.edu/ or at GEO under accession number GSE15523.

### Microarray data analysis

Elements on the array flagged as “spot not found” or “spot bad” were removed from data and not considered further. The background subtracted LOWESS (for time course data) or global linearly (steady state experiment) normalized data were filter for spots with intensity at least 20% above background. In each time course series (LCPX, MYCN-ER, or MYCN(ΔMBII)-ER), we required the data for at least two of the three zero time points (for a given probe) to pass the above criteria or the gene was removed from further consideration. Similarly for the steady state experiments, we required the data for both vector arrays for a given probe to meet the above criteria or the gene was discarded. Subsequently, only those probes that met the above criteria for at least 80% of the 25 arrays in the time course experiment or of the 6 arrays in the steady state experiment were retained.

For a given probe in a time course series, the average expression value across the three zeros was subtracted from every expression value in that series. A similar zeroing was performed on the steady state data except the vector expression values served as the zeroes. In the time course data, genes that showed at least a 1.7 fold change from the time zero point, in at least two arrays were selected for further analysis. The probes (1631 probes) that pass these filters for the time course experiment were considered MYCN-ER responsive. SAM (Significance Analysis of Microarrays, version 1.21) was used to perform a two-class, unpaired analysis of the steady state data with the wild type MYCN arrays in one class and the vector and mutant MYC arrays in another [35]. The resulting probes (1608 probes) from this analysis were considered MYCN responsive. Hierarchical clustering analyses were performed with Cluster 3.0 [36].

The Core MYC signature centroid was calculated in three steps. First, the average expression values for the Core MYC signature genes across the last three time points (24, 36, and 48 hrs) in the time course experiment were determined. Second, the average expression values for the Core MYC Signature genes across the duplicate MYCN arrays were determined. Finally, the average of these two averages resulted in the Core MYC Signature centroid. Therefore, the MYCN-ER induction and MYCN overexpression studies equally contribute to defining the Core MYC signature centroid.

When different expression profiling datasets were linked together, publicly available datasets were first filtered, normalized and centered as in the original study. Then, platform-specific clone identifiers were converted (using Source and other utilities) to UNIGENE cluster identifiers (unigene build 219, June 2009). Data from multiple spots mapping to the same UNIGENE cluster ID were averaged across the multiple spots. The data were then linked by unique UNIGENE cluster IDs.

For all data presented as Java Treeview images, the contrast setting was set to “2”.

Classification of breast tissue samples from the Hu et al. and the NKI295 datasets was based on the SSP/Spearman classification system described in Hu et al. [37].

### Immunohistochemistry

Paraffin-embedded breast tumors were cut into 5 µm sections. Tissue sections were deparaffined with xylene, dehydrated with ethanol and endogenous peroxidase activity was blocked with a 3% hydrogen peroxide solution. The slides were incubated with 10 mM citrate buffer (pH 6.0) and microwaved for 20 min for antigen retrieval. The slides were then blocked with goat serum and incubated with cMYC antibody for 30 min (Santa Cruz sc-40, 1∶50 dilution), and then incubated with biotin conjugated goat anti-mouse IgG (Vector Laboratories, CA, USA). Proteins were visualized with streptavidin-conjugated HRP (Vector Laboratories, CA, USA). The slides were counterstained with 50% hematoxylin and examined by light microscopy on a Zeiss microscope at×100 magnification.

## Results

### Experimental design

The pathology that results from MYC overexpression is the consequence of altered expression of both direct and indirect target genes. Hence, we designed our experiments to capture the complete cellular transcriptional response to MYC overexpression rather than try to limit our study to direct MYC target genes. Experiments to identify MYC responsive genes were performed with human primary foreskin fibroblasts harboring inducible or constitutive MYC constructs. We chose to examine MYCN as a model member of the MYC family of proteins. MYCN is a MYC family member that acts similarly to MYC in most experimental systems [34], [38] and in gene response profiles (Cowling and Cole, manuscript in preparation). In addition to wildtype MYCN, we studied a mutant MYCN with a deletion within MYC homology box II (MBII), a conserved region among MYC family members shown to be essential for many of MYC functions such as the regulation of some target genes, transformation, proliferation, and apoptosis [34], [39]–[41].

We created both inducible and stable expression vectors for MYCN. MYCN-ER^TM^ was created as a fusion protein between a full length mouse MYCN cDNA and a portion of the estrogen receptor [33]. We also used a constitutive MYCN vector for stable, increased expression [34]. The exogenous inducible and constitutive constructs were introduced into primary human foreskin fibroblast cells by retroviral transduction, and expression from the exogenous transgenes was confirmed by western blot (Supplemental Figure S1). Genome-wide gene expression was then analyzed using oligonucleotide microarrays.

### Overview of MYC induced Expression Profiles

**Figure 1:**
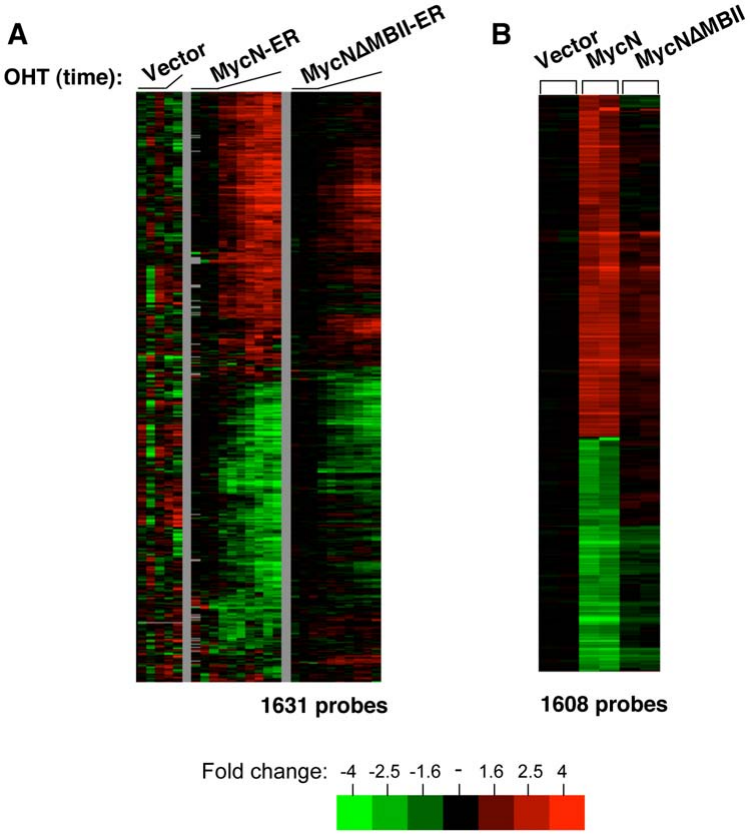
Microarray data of MYCN responsive genes. A) Probes from the MYCN-ER time course experiment after clustering. Probes were selected as described in Methods and the expression data was subjected to hierarchical clustering. B) Probes from the MYCN steady state expression studies after selection and hierarchical clustering. Complete lists of probes/genes are provided in Supplemental Table S1.

For MYCN-ER^TM^, cells were expanded after viral transduction and treated with 4- hydroxytamoxifen (4-OHT). Total RNA was harvested in a time course. Untreated zero time points from three biological replicates of the lines (vector, MYCN-ER^TM^, MYCNΔMBII-ER^TM^) were collected. Single vector control samples were collected 8 and 48 hr following induction with 4-OHT. Single samples of MYCN-ER^TM^ and MYCNΔMBII-ER^TM^ were collected 2, 4, 8, 12, 24, and 48 hr following induction. Microarray analysis was performed on collected samples. Genes that showed at least a 1.7 fold change from the time zero point in at least two arrays were selected for further analysis. Data from 1,631 probes (representing 1,449 unique UNIGENE clusters (Build 219) that pass these filters for the time course experiment were considered MYCN-ER responsive (Supplemental Table S1). After filtering, the genes were organized by one-dimensional hierarchical clustering (Figure 1A). To confirm array results, eight randomly selected genes among the MYCN responsive genes were examined by RT-PCR. Transcripts for all eight genes showed similar patterns by both RT-PCR and microarray analyses (data not shown).

Comparison of the gene expression profiles between MYCN-ER^TM^ and MYCNΔMBII-ER^TM^ provides insight into the transcriptional defect in the latter mutant. MYCNΔMBII-ER^TM^ is severely blunted, but not completely defective, in the number and magnitude of both gene activation and repression (Figure 1A). A similar conclusion was previously reached from the steady state data[42]. The defect in activation and repression was roughly equivalent. The genes induced or repressed by MYCNΔMBII-ER^TM^ mutant were also generally delayed in response compared to wt. A subset of genes are still induced and repressed by the MYCNΔMBII-ER^TM^ mutant but further analysis will be required to determine if these promoters have any unique features.

For comparison to the time course data, the same MYCN and MYCNΔMBII proteins were stably overexpressed in BJ fibroblasts, an experimental design that more closely approximates the situation in tumors that have high MYC expression. Cells were virally transduced with the constitutive MYCN expression constructs, stably expressing cells were selected, and total cellular RNA was harvested. Technical duplicates for vector, MYCN, and MYCNΔMBII cells were assayed for differentially expressed genes using the same human oligonucleotide microarrays used for the time course. Expression from the exogenous transgenes was confirmed by western blot [42]. These data have been previously presented in the context of another study [42], but will be discussed here in comparison to the time course data and with different statistical tools.

We used SAM (Significance Analysis of Microarrays, version 1.21) to perform a two-class, unpaired analysis with the wild type MYCN arrays in one class and the vector and mutant MYCN arrays in another [35]. SAM analysis was used to focus attention on genes induced specifically by wildtype but not mutant MYCN. SAM analysis identified 1608 differentially expressed probes with a median false discovery rate of 0.42%. These 1,608 probes map to 1,407 unique UNIGENE clusters (Build 219) (Supplemental Table S1). The identified steady state MYC responsive probes were clustered hierarchically using Cluster 3.0 (Figure 1B).

### Comparison to the “MYC Target Gene Database”

**Figure 2:**
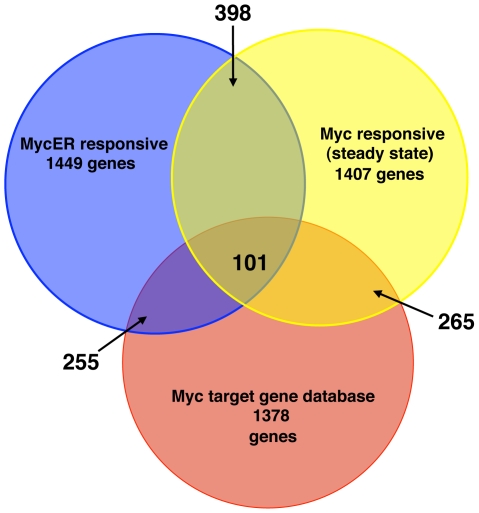
Venn diagram of MYC responsive genes in different studies. MYC responsive genes in our studies and those found in the MYC cancer database were mapped to unique unigene cluster ID's. The total number of target genes from the MYC database is the number of genes in the database that map to unique unigene cluster ID's and that had a probe on the Agilent arrays.

Comparison of our study to previous studies that identified MYC responsive genes underscores both the magnitude of the MYC response as well as the differences inherent to the experimental approaches. To be as inclusive as possible for comparisons, we considered the MYC Target Gene database (http://www.myccancergene.org/) as a repository of previously described MYC responsive genes [10]. The MYC Target Gene database includes MYC responsive genes identified in independent studies by SAGE, DNA microarray, subtractive hybridization, ChIP, northern/western blot or RT-PCR. To compare the MYCN-ER responsive genes and the steady state MYCN responsive genes with a current compilation of MYC targets in the MYC Target Gene database, we mapped MYCN responsive genes identified here, and those found in the MYC cancer database to UNIGENE cluster IDs. The total number of target genes from the MYC database is the number of genes in the database that map to a unique UNIGENE ID (UNIGENE Build 219) and that were represented on the Agilent microarrays (1,378 genes). Similarly, the number of MYCN-ER responsive (1,449 genes) or MYCN responsive genes (1,407 genes) is the number of responsive probes that map to unique UNIGENE clusters (UNIGENE build 219). In Figure 2, we compare these data in a Venn diagram. When individually compared to the MYCN database, the MYCN-ER responsive genes overlap with only 255 genes out of 1,378 database genes and the steady state MYCN responsive genes overlap with only 265 out of 1,378 database genes. Surprisingly, only 101 genes are common to all three groups (all genes in this analysis are provided in Supplemental Table S3). Thus, our analysis reveals a very large set of previously unrecognized MYC responsive genes. It is important to add that this lack of overlap among MYC responsive genes is consistent with other analyses of expression and computationally derived gene sets [31].

### Definition of the Core MYC signature

**Figure 3:**
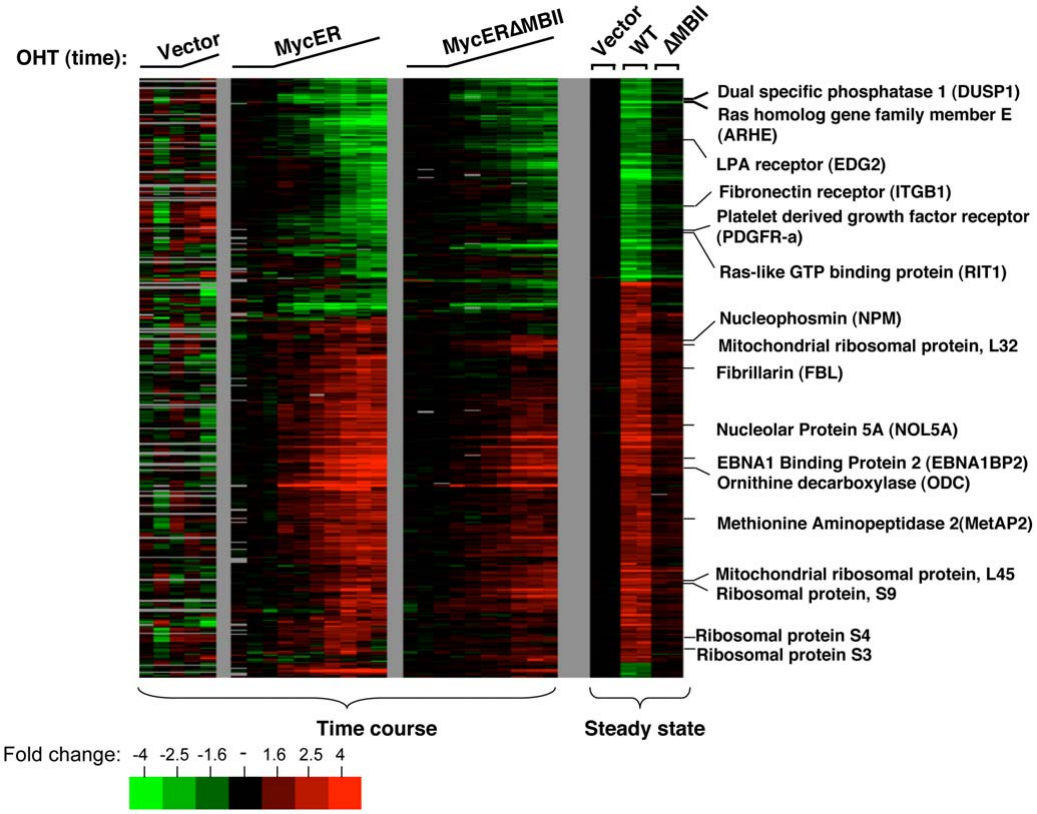
The Core MYC gene expression signature. Expression data for probes that are identified as responsive to both MYC-ER activation and steady state MYC overexpression are hierarchically clustered. The expression response of these probes is defined as the Core MYC signature. The signature probes total 428 Agilent probes that can be mapped to 385 unique unigene clusters.

The two different data sets from induced and steady state MYCN overexpression suggest that the response to MYCN overexpression can be quite variable, depending on the method and timing of delivery. We reasoned that the genes common to the MYCN response in both experimental approaches would serve as a conservative estimate of a core ‘MYC gene expression signature’. Thus, we chose to focus on the genes responsive to MYCN overexpression in both experimental designs. Here forward we refer to this expression profile as the ‘Core MYC Signature’ (Figure 3).

The Core MYC Signature consists of 428 probes that map to 398 unique UNIGENE clusters (Build 219) (Figure 3, Supplemental Table S1). Of these genes, 354 (89%) have the same direction of response in both studies (Figure 3). We assessed the GO term annotations for the clusters of Core MYC signature genes that display a common direction of response and found that the induced genes and repressed genes have a very similar set of overrepresented GO Terms to that found in the individual studies described below (see below, Supplemental Table S2).

The different experimental designs dictated different methods of categorizing genes as MYCN responsive. We were interested in further examining the genes classified as responsive in one experimental design, but not the other. To do this, we examined the expression data of the probes in the non-overlapping areas of the Venn diagram in Supplemental Figure S2. We noticed that a substantial number of these non-overlapping genes were apparently responsive in both studies. Further analysis shows that while many of theses genes do respond in both studies, the response is weak or noisy in one study thereby not passing the previously decided criteria for MYCN responsiveness. Lowering the thresholds for MYCN responsiveness leads to a larger group of overlapping genes. One explanation for these differences could be the kinetics of target gene response. MYCN-ER is activated transiently for a few hours whereas the steady state cells took three weeks of continuous culture to expand the polyclonal population. Short term versus long-term response to high MYCN levels could alter the magnitude and spectrum of the gene expression profiles.

### MYCN responsive genes

Gene Ontology (GO) terms allow gene lists to be interrogated for the overrepresentation of specific biological processes. Gene Ontology TermFinder (version 0.61) is a statistical tool (with multiple hypothesis testing correction) to measure the overrepresentation of GO terms in clusters of similarly responding genes [43]. The enrichment of GO terms among the clusters of MYCN responsive genes provides an unbiased assessment of specific biological processes that are nonrandomly represented in each cluster. Analysis of GO term enrichment in clusters of genes that are regulated by MYCN-ER, MYCN and also those in the Core MYC Signature reveals many significantly overrepresented GO Biological Process terms. For induced genes, there is a striking overrepresentation of genes involved in translation, protein biosynthesis, ribosome biogenesis and assembly, RNA processing, and nucleotide metabolism (Table 1, Supplemental Table S2). In the long-term model of MYCN overexpression, the GO terms cell proliferation and DNA replication are also overrepresented (see Supplemental Table S2 for p-values). These data are consistent with several previous reports identifying similar links between protein synthesis and MYC, strongly suggesting that MYCN overexpression results in an increase in general protein synthesis.[44], [45]. Genes involved in ribosome biogenesis such as FBL (Fibrillarin), NPM1 (Nucleophosmin 1), NOL5A (Nucleolar protein 5A) and EBNA1BP2 (EBNA1 binding protein 2B) are upregulated by MYC overexpression (Figure 2). Similarly, genes that encode products more directly involved in protein synthesis such as 60S ribosomal protein L32, 39S ribosomal protein L45, 40S ribosomal proteins S3 and S4 (Y isoform), 28S ribosomal protein S9 and MetAP2 (Methionine aminopeptidase 2) are also upregulated (Figure 2).

**Table 1:** Representative subset of enriched GO terms among induced MYC signature genes.

Enriched GO terms among induced MYC signature genes	P-value (Bonferroni-corrected)
RNA processing	0.00E+00
RNA metabolism	1.16E-09
ribosome biogenesis	4.96E-09
rRNA processing	4.56E-06
mRNA processing	1.53E-04
RNA splicing	1.16E-03
regulation of translation	7.21E-03
protein biosynthesis	7.51E-03

Enriched GO terms among the repressed genes revealed a striking trend. Genes involved in development, signal transduction and cell communication are repressed (Table 2 and Supplemental Table S2). A common element in these biological processes is that each involves the cell's ability to recognize and communicate external cues to the nucleus. Genes such as Rit1 (Ras-like GTP-binding protein), STAT1 (Signal transducer and activator of transcription 1), EDG2 (LPA receptor), ITGB1 (Fibronectin receptor, Integrin beta 1), SOS-1 (Son of sevenless protein homolog 1) are repressed and involved in these processes. Finally, among the repressed genes, we found that there was an overrepresentation of genes whose products are extracellular (p<3.8×10^−8^). (Supplemental Table S2).

**Table 2:** Representative subset of enriched GO terms among repressed MYC signature genes.

Enriched GO terms among repressed MYC signature genes	P-value (Bonferroni-corrected)
development	1.54E-04
blood coagulation	1.81E-03
wound healing	2.36E-03
hemostasis	2.58E-03
signal transduction	2.76E-03
cell communication	4.12E-03
response to external stimulus	1.31E-02

Several attributes of the list of repressed genes are notable. First, the number of repressed genes forms a significant portion of the complete transcriptional response. In the MYCN-ER experiments, approximately 48% of the response corresponds to repressed genes. In the MYCN steady state experiments, approximately 41% of the MYC response is composed of repressed genes. Second, a substantial fraction of the repression response initiated by MYCN-ER activation occurred as rapidly as the earliest induction response. Rapid repression will only be observed for genes with rapid mRNA turnover, so repressed genes may be underrepresented in the early points of a time course. For example, PDGFR-alpha peptide (platelet derived growth factor receptor, alpha peptide 1), DUSP1 (dual specific phosphatase 1) and ARHE (Ras homolog gene family member E) show a repression response after only two hours of MYCN-ER activation (Figure 3). Third, as discussed above, GO term enrichment analysis shows that many of the repressed genes appear to be involved in communicating to other cells or in responding to extracellular signals (Table 2 and Supplemental Table S2).

### Analysis of MYC binding sites in Core MYC signature genes

To gain insight into which of the Core MYC signature genes are likely to be direct targets, we compared the gene set to two recently published studies that used chromatin immunoprecipitation to define genome-wide MYC binding sites in embryonic stem (ES) cells (see Supplemental Table S5 for full comparisons) [46], [47]. Of the total 398 MYC signature genes, 146, or 36.7%, have direct binding sites mapped by Kim et al.(2008) or by Kidder et al.(2008) Interestingly, of the Core MYC Signature genes that are induced by MYCN-ER or by MYCN, 134/254 (52.8%) have direct binding sites mapped by Kim et al.(2008) or by Kidder et al. In stark contrast, of the Core MYC Signature genes that are repressed by MYCN-ER or by MYCN, only 22/164 (13.4%) have direct binding sites mapped by Kim et al.(2008) or by Kidder et al. See Supplemental Table S6 for full enumeration of lists in Table 3. These data are consistent with established mechanisms of MYC activity - activated genes usually have MYC bound proximal to the promoter whereas repressed genes rarely, if ever, have canonical MYC binding sites [48]. Despite the sizeable fraction of MYCN/MYCN-ER induced genes having evidence for direct MYC binding, this analysis is a comparison of fibroblast expression data to embryonic stem cell MYC binding data. Therefore, there is a strong likelihood that genes that are regulated in the MYC signature may indeed have MYC bound at their promoters, but are simply not regulated and bound in ES cells.

**Table 3:** MYC-bound Core MYC signature genes.

Induced MYC Signature genes	254
That have a direct MYC binding site	134
**Percentage**	**52.8%**
Repressed MYC Signature genes	164
That have a direct MYC binding site	22
**Percentage**	**13.4%**

The regulated genes in this table include MYC Signature genes that are regulated in the indicated fashion by either MYCN-ER or by MYCN. The genes that are indicated as having direct MYC binding sites are those identified in the Kim et al.(2008) or Kidder et al. study.

### Analysis of the Core MYC Signature in a serum response dataset

**Figure 4:**
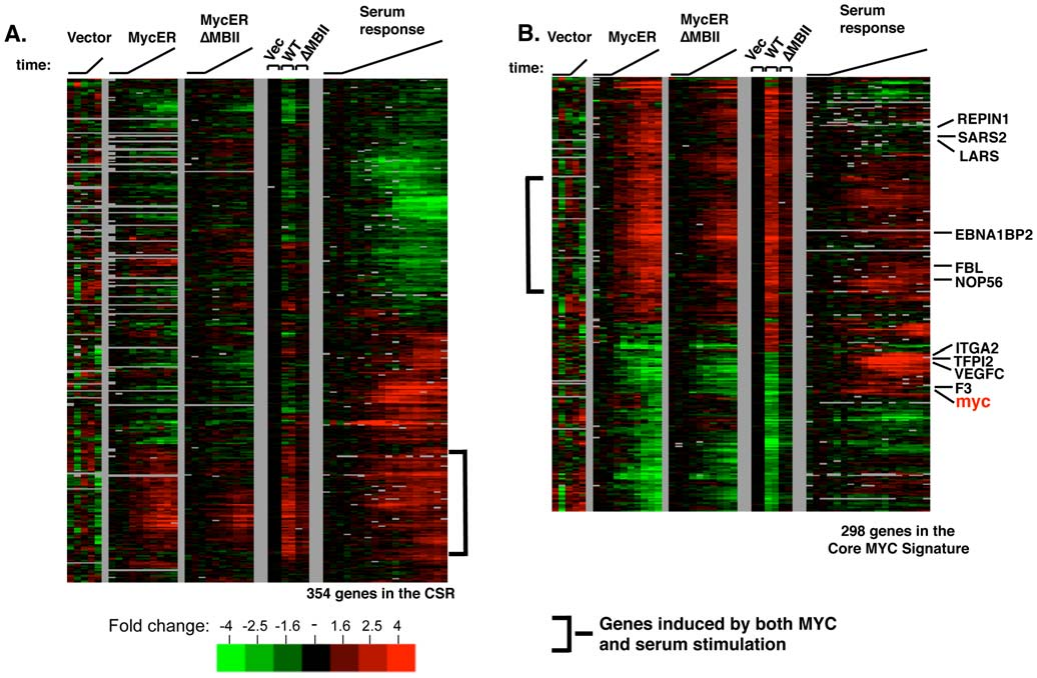
Serum stimulation vs. MYC overexpression. A. The expression profiling data for serum responsive study [20] and this study were linked. The data corresponding to the (CSR) gene list were extracted from the linked dataset. The data for these genes were hierarchically clustered in one dimension (genes). Upon serum stimulation, samples were harvested from 0 hrs to 36 hrs. B. The same procedure was performed starting with the Core MYC signature gene list. The data for each of the Core MYC signature genes was extracted from the linked dataset and clustered in one dimension.

The c-MYC gene was the first oncogene shown to be part of the immediate early response to serum stimulation [16]. Therefore, it was of interest to compare the response of fibroblasts to MYCN overexpression with their response to serum. We utilized a publicly available serum stimulation gene expression dataset of normal human fibroblasts, and a list of genes determined to represent the “Core Serum Response” (CSR) [20]. *A priori*, we expected that MYCN overexpression might induce a gene expression response that was similar to the serum response. Specifically, we thought that MYCN would induce genes that are called delayed early response genes. Delayed early response genes are defined as those that are dependent on the initial induction of a transcriptional regulator or signaling pathway. This initial transcriptional response subsequently induces a secondary set of genes that are not induced by the primary signal. These expectations were met for a fraction of the CSR genes that could be mapped to our array platform (Figure 4A). A set of genes (80 out of 354; 23%) is induced both by MYCN and by serum, and the kinetics of induction in the CSR are consistent with these genes being downstream of MYC. In the serum induced gene profiles, the c-MYC gene itself is induced at 2–4 hr, similar to the response described in numerous studies. However, in the MYCN-ER and steady state overexpression profiles, the c-MYC gene itself scores as repressed due to autoregulation. It is important to add that although there is an overlap between the CSR and MYCN induction, many of the CSR genes would not have passed the threshold as MYC targets in either the kinetic or steady state experiments. Not surprisingly, many CSR genes are not MYCN responsive, implying that these genes are regulated by MYC independent pathways or require some combinatorial signal.

A somewhat different pattern emerged when we analyzed the Core MYC signature gene set for their response to serum (Figure 4B). 298 genes of the Core MYC signature genes were represented on the arrays of the serum response study. 30 of these genes are found in the CSR. Of these 30 genes, 22 of the Core MYC signature genes displayed a concordant response in the CSR, i.e. were induced or repressed the same. The concordant 22 genes of the 298 Core MYC signature gene set represents a statistically significant overlap (p-value<8.6×10^−5^). These 22 genes (7.4%) were induced or repressed by serum with kinetics consistent with them being MYC-dependent delayed early genes (Supplemental Table S4). Interestingly, many of the concordant genes consistently upregulated by both MYCN and serum are involved in formation and maturation of ribosomes (FBL, EBNA1BP2, NOL5A) [45], [49], [50]. In contrast, greater than 50% of the Core MYC Signature genes were not induced or repressed by serum at all (i.e. REPIN1, LARS, SARS2). Some genes induced or repressed by MYC exhibited the opposite response to serum (i.e. TFPI2, ITGA2, VEGFC, F3) (Figure 4B). Expanding the analysis to the larger sets of genes responsive to MYCN-ER or steady state MYCN does not appreciably change the percentage overlap (data not shown). Thus, MYCN induces a large set of genes that are not significantly induced by normal serum growth factors. For a summary of the comparisons, see Supplemental Table S4.

### Systematic analysis of the Core MYC Signature in breast tumors

**Figure 5:**
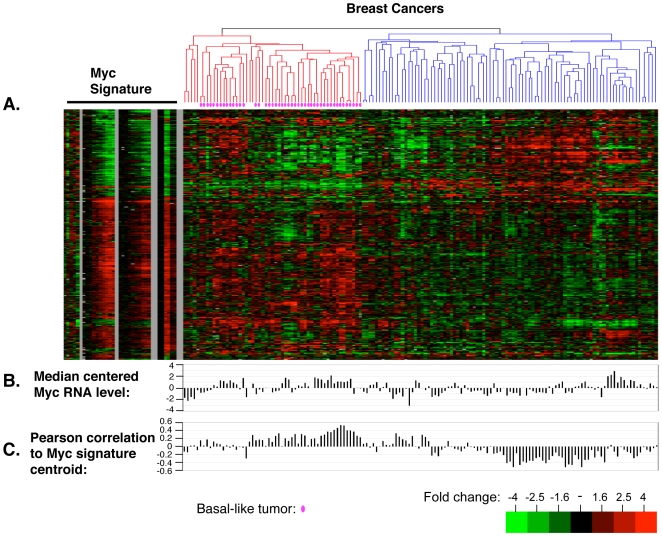
The MYC gene expression signature in breast tumor tissue. A. The Core MYC signature gene list was extracted from a breast cancer profiling dataset containing 105 tumor samples, 9 normal breast samples, and 26 sample pairs [37]. The data for these probes were hierarchically clustered in two dimensions (genes and arrays). The order of breast tissue arrays (right) was preserved before linking to the Core MYC Signature data (left). The merged data set was hierarchically clustered in one dimension (genes only) to give the presented format. B. The median centered c-MYC RNA levels were extracted from the breast tissue expression profiling study. C. Pearson correlation between the Core MYC signature and the breast cancer profiles.

We next assessed the expression of the Core MYC signature in the pathophysiological settings of tumors. Since MYC is frequently amplified and overexpressed in breast cancers, we extracted the genes of the Core MYC signature from expression profiles of a set of 146 breast tissue samples that were previously hybridized to the same Agilent human oligonucleotide microarrays [37], and we determined if the Core MYC signature is found in any of the previously defined subtypes of breast tumors. We first used the Core MYC signature gene list to cluster the breast tumor samples hierarchically in two dimensions, which groups the tumors into two main clusters (Figure 5). To examine the robustness of the sample groupings, we used Significance of Clustering (SigClust) [51]. We find the two major bifurcations in the dendrogram tree are significant (p<0.001). The left (red) branch of the dendrogram includes all of the basal-like tumors and only nine non-basal tumor samples (seven are HER2+/ER− tumors and two are luminal-type tumors) (Figure 5A, Table 4). The right dendrogram branch is not composed of one predominant subtype as was seen for the left cluster, and contained a mix of Luminal, HER2+, and Normal-like samples (Table 4). The distribution of tumor subtypes in the two clusters suggests a statistically significant relationship between the cluster grouping and subtype (using a chi-square analysis) (Table 4).

**Table 4:** Hu et al. samples clustered by the Core MYC Signature.

	Left	Right	Total
**Basal**	46	0	46
**HER2+/ER−**	7	16	23
**Luminal**	2	60	62
**NB**	0	15	15
**Total**	55	91	146

Degrees of freedom: 3.

Chi Square = 117.

p-value<0.001.

The distribution of breast sample subtypes in the clusters presented in the hierarchical clustering in Figure 5. Colored dendrogram branches from Figure 5 correspond to the colors used in this figure. LumA, Luminal type A. LumB, Luminal type B. NB, normal breast.

The observation that the genes of the Core MYC signature can drive the grouping of the basal tumors suggests that MYC itself may be coordinately expressed with the signature in this subtype. To explore relative differences in MYC RNA levels across the tumors, we displayed the median centered data for MYC in a histogram below the tumor samples (Figure 5B). Among the tumor samples, the basal tumors have the highest average MYC levels. Furthermore, the general expression pattern (induced or repressed) of Core MYC signature genes in the basal tumors most closely parallels the response of these genes to ectopic MYCN expression in primary fibroblasts. Interestingly, some of the normal breast tissue samples that cluster on the right (blue) branch of the array dendrogram have relatively high MYC expression but display an expression profile that is clearly distinct from the samples in the left cluster of mostly basal-like tumors.

### Detecting the presence of the Core MYC signature

To quantify the relationship between the Core MYC signature response and the expression profile of an individual tissue, we first calculated a Core MYC signature centroid to which the resemblance of each expression profile is calculated as a Pearson's correlation. A similar strategy was previously employed to calculate the presence of the “wound signature” in tumor expression profiles [52]. In our study, we use this correlation value as a means to describe the presence of the Core MYC signature in a given sample. However, it is difficult to decide a threshold correlation value above which one would classify a sample as definitively displaying the Core MYC signature. For comparison, the absolute value of a correlation score describing the presence of the wound signature in a tumor sample of greater than 0.15 had substantial predictive clinical consequences [52]. The Pearson correlations between the MYC centroid and breast tumor expression profiles are presented in Figure 5C. The left and right clusters display average correlation scores of 0.16, and −0.13, respectively. In the left cluster, most tumors had a positive correlation score and some tumors had correlation scores greater than 0.5.

**Figure 6:**
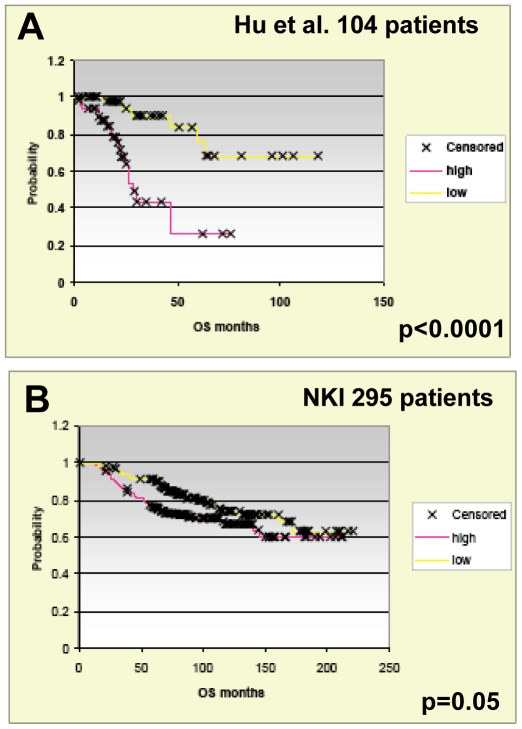
Kaplan Meier survival analyses in the Hu et al. and NKI datasets. Patients from the Hu et al. (A) and the NKI295 (B) datasets were grouped by low/high Core MYC signature average expression. Survival analyses were performed on patients from these groupings.

To more objectively search for associations between the Core MYC signature and tumor subtype, we created an “average expression profile” for each patient across the Core MYC signature genes by simply deriving an average expression value across all Core MYC signature genes for each sample. Next, the patients were put into rank expression order based upon this average Core MYC signature expression value. The patients were then put into either the “low” or “high” Core MYC signature expression groups based upon a 50/50 split of the samples. Using chi-squared analyses where the distinction of high vs. low is compared to the classification of intrinsic subtype in the Hu et al. set of 146 samples analyzed, a statistically significant relationship between the Core MYC signature average expression and subtype was observed (Table 5); the higher average expression was most commonly seen in the basal-like tumors that frequently show 8q24 amplification [53], and the lower average expression was observed more often in the good outcome Luminal A subtype. Similar results were obtained when an identical analysis was performed on the NKI295 sample set of van't Veer et al., and van de Vijver et al. [54], [55] (Table 6). Lastly, using this Core MYC signature average profile and the distinction of “high” vs. “low” expression, we conducted Kaplan Meier survival analyses in both the Hu et al. and van de Vijver et al. data sets, which suggested that this profile by itself may be of prognostic significance (Figure 6). The classification of tumor samples into “high” vs. “low” expression yielded starkly different survival curves in the Hu et al. data set, while the differences in survival curves in the NKI295 sample set were significant, yet decidedly more subtle. The reason for this difference in the two data sets is likely due to the difference in microarray platforms used in these studies. The Hu et al. study utilized the identical microarray platform as this MYC study permitting greater overlap of genes for subsequent survival analysis. The NKI295 samples sets were analyzed on a different microarray platform that provided a reduced set of overlapping genes for the survival analysis.

**Table 5:** Hu et al. sample subtypes sorted by low/high Core MYC Signature average expression.

	Left	Right	Total
**Basal**	14	32	46
**LumA**	28	13	41
**LumB**	13	8	21
**HER2+/ER−**	11	12	23
**NB**	7	8	15
**Total**	73	73	146

Degrees of freedom: 4.

Chi Square = 13.8.

p-value<0.01.

The distribution of Hu et al. breast sample subtypes in the low and high Core MYC signature grouping. LumA, Luminal type A. LumB, Luminal type B. NB, normal breast.

**Table 6:** NKI295 sample subtypes sorted by low/high Core MYC Signature average expression.

	Left	Right	Total
**Basal**	9	44	53
**LumA**	80	43	123
**LumB**	32	23	55
**HER2+/ER−**	13	22	35
**NB**	13	16	29
**Total**	147	148	295

Degrees of freedom: 4.

Chi Square = 38.3.

p-value<0.001.

The distribution of NKI295 breast sample subtypes in the low and high Core MYC signature grouping. LumA, Luminal type A. LumB, Luminal type B. NB, normal breast.

### Immunohistochemical analysis of MYC in breast tissues

To more thoroughly examine the relationship between tumor subtype and MYC expression we performed immunohistochemistry for MYC protein expression in a subset of the tumors presented in Livasy et al. [56]. This sample set represents a group of breast tumors that were analyzed using RNA recovered from fresh frozen material and for which paraffin-embedded materials were also available for protein expression studies. We performed IHC on 26 tumors in total, which included 11 Basal-like, 3 HER2+/ER− and 12 Luminal/ER+ tumors using antibodies against MYC. Representative IHC images of a basal-like tumor and normal breast tissue are shown in Supplemental Figure S3. Ten out of eleven of the basal-like tumors were positive for MYC, as were two out of three HER2+/ER−. In contrast ten out of twelve Luminal/ER+ tumors were negative for MYC. As was seen in the gene expression studies, significant expression of MYC was also seen in normal breast epithelial tissue (see Figure 5). These protein expression results corroborate the gene expression results for MYC itself and support the conclusion that the MYC gene expression signature is a common, and perhaps critical, characteristic of basal-like breast tumors.

## Discussion

The fact that nearly all mammalian cell proliferation is contingent on the expression of at least one MYC family protein hints at the central role that MYC plays in normal growth. Given its critical role in proliferation, it is not surprising that aberrant or deregulated MYC expression participates in the malignant transformation of normal cells into cancer cells. Numerous high-throughput studies querying MYC regulated genes have been published [22]–[29], [57]. Perhaps surprisingly, little overlap in target genes is found among all the studies. A trivial but important reason for part of the non-overlap is that the genes on the microarrays were different from study to study. As newer microarray platforms and approaches have permitted complete or near complete genome wide inquiry, this problem may be resolved. Numerous other possible explanations stem from methodological differences including choice of cell type, the levels of MYC overexpression, time of overexpression, and the choice of MYC family member as the exogenous transgene. We show here that the response to MYC can vary significantly even in a single cell type (primary human fibroblasts) depending on the timing and dosage of MYC delivery (Supplemental Figure S2). The use of an estrogen receptor offers the advantage of a rapid kinetic response, yet the artificial nature of the fusion protein may alter the target gene response compared to wt MYC. On the other hand, constitutive MYC expression may induce a broad change in the transcriptional and posttranscriptional program that includes many indirect effects. We sought to overcome the limitations of each technique by focusing on genes that responded to MYC induction in two independent protocols within the same cell type. Both protocols show that MBII is critical for the full induction and repression of the majority of MYC responsive genes.

After defining a Core MYC signature, we compared the transcriptional response in fibroblasts between MYCN overexpression and serum stimulation. This is of particular relevance because MYC is an immediate early serum response gene, i.e. induced by serum stimulation even in the absence of protein synthesis. We anticipated that a significant fraction of the Core MYC signature would be part of the serum response, perhaps focused on a ‘delayed early’ gene set that is dependent on prior MYC expression. Furthermore, previous studies have shown that overexpression of MYC can bypass the need for serum growth factors. We were surprised to find that only a fraction of the Core MYC signature (10%) falls within the core serum response gene set, which was also derived from primary human fibroblasts [20]. Furthermore, the response to MYC and serum is sometimes in opposite directions for individual genes. Nevertheless, 23% of the induced CSR genes are also MYC induced (Figure 4A). There are at least three possible explanations for this observation (which are not mutually exclusive). First, serum is a heterogeneous stimulus that is likely to activate many different pathways. The final expression levels of genes may be the consequence of the additive or competing regulatory influences of different pathways. Hence, while serum stimulation induces MYC expression, which may alone lead to the activation of a gene, other serum activated pathways may lead to a repressive regulatory influences on that same gene. Second, deregulated MYC expression afforded by the viral LTR and/or ER domain may exceed the level of MYC protein induced by serum. MYC/Max binding to promoter sites is dependent on expression level, with more sites occupied at higher MYC levels [58]. However, it is not yet clear if elevated levels of MYC lead to novel responsive genes or simply an exaggerated response. Third, experimental differences in this study and the CSR study may account for differences observed in the Myc signature and the serum response. Some serum regulated genes (which are also Myc regulated) may have achieved their maximum response before Myc was overexpressed in our system since cells were not serum starved before MYCN-ER activation. However, the bulk of the Myc signature genes (genes whose transcripts do change with MYCN overexpression in the presence of serum) do not have the cognate response to serum (Figure 4B). It is clear that the cellular response to MYC alone is qualitatively distinct from a serum response, with a wide range of genes activated and repressed that are normally unaffected by serum growth factors. A previous study reached a similar conclusion that MYC induction and serum stimulation were distinct using a B cell model [59].

While many studies have focused on the discovery of MYC target genes, few have examined the genome-wide transcriptional changes induced by MYC. A recent study of gene expression in a murine model of prostatic intraepithelial neoplasia (mPIN) created by targeting MYC overexpression to the prostate has defined a prostate cancer specific MYC induced gene expression signature [60]. In another study, analysis of gene expression profiles of neuroblastomas identified coordination between high MYCN levels and high levels of another oncogene, H-Twist, that functions to antagonize MYCN's proapoptotic effects [61]. Recently, Adler et al. show that the underlying drivers of the wound signature in human breast cancer were the amplification of MYC and CSN5 [21]. The wound signature is most robustly recapitulated by the overexpression of both MYC and CSN5. In most of these studies, comparison between normal tissues with low MYC levels to tumor tissues with high MYC levels was used to define the MYC dependent gene expression profile. Because the comparisons are to tumors with high MYC expression that are likely to have suffered multiple other genetic lesions, these strategies reveal the transcriptional profile after all genetic lesions have occurred, not just those associated with MYC activation alone. Since the Core MYC signature defined in the present study includes both kinetic data and an enrichment for MYC/Max binding sites, it is more likely to contain direct MYC targets. A fundamental MYC gene expression profile may be broadly relevant to different types of MYC-driven tumors and should facilitate elucidation of the complex cellular responses that mediate the biological activities of the MYC protein.

A key finding of this study is the distinct correlation between the Core MYC signature and tumors of the basal-like subtype of breast cancer. Our IHC data corroborate the association between high MYC expression and the basal-like tumor subtype, and this strongly suggests a common underlying biological program in effect in both the basal-like tumors and the MYC induced fibroblast cells. From these data it is not formally possible to conclude that expression of the Core MYC signature orchestrates the typically aggressive behavior of the basal-like breast tumors; however, it is likely that this expression profile is not inconsequential. In support of this direct link is the finding that MYC mRNA expression and the Core MYC signature are linked to basal-like tumors in two different patient data sets, and in both bases, the Core MYC signature is also of prognostic value. Further support for a link between MYC and basal-like breast cancers comes from studies of the transformation of human mammary epithelial cells (which are basal-like cell lines [62], [63]) with hTERT, SV40 T-antigen and H-Ras. Transformation was frequently accompanied with the amplification of MYC, suggesting that even in the presence of multiple other oncogenes, amplification of MYC is still required to transform this basal-like cell type [64]. Furthermore, high levels of MYC can partially transform immortalized human mammary epithelial cells [65]. A recent meta-analysis study further supports elevated MYC and E2F activity in the basal-subtype tumors [66]. Lastly, it has also been shown that MYC is amplified in many BRCA1-associated tumors [67], which are known to be mostly basal-like tumors [68]. Thus, basal-like mammary epithelial cells are exquisitely sensitive to MYC overexpression, and it is likely that MYC is a significant etiological factor in the development of this subtype of breast tumor.

In conclusion, using two different cell culture models of aberrant MYCN expression, we have defined a fundamental gene expression profile that is less subject to many of the common experimental variables. The Core MYC Signature is distinct from the Core Serum Response, yet the two profiles share statistically significant gene expression responses. The Core MYC Signature has clinical relevance as this profile is identified in the basal-like subtype of breast cancer expression profiles, which typically harbor the highest levels of MYC expression among the different breast cancer subtypes. Importantly, the Core MYC signature can be used to deduce an underlying genetic program that is likely to powerfully contribute to a clinical phenotype. Therefore, the presence of the Core MYC Signature may predict clinical responsiveness to drugs that are designed to disrupt MYC-mediated phenotypes.

## Supporting Information

Figure S1 Analysis of expression from transgenes Whole cell lysates of primary human fibroblast cells infected with pLPCX (lane 1) pLPC-MYCN-ER (lane 2), and pLPC-MYCN(del-MBII)-ER (lane 3) were resolved by PAGE and transferred to PVDF for western blot analysis. Because exogenous proteins were FLAG-tagged, western analysis was performed using antibodies specific to the FLAG epitope.
(0.19 MB TIF)

Figure S2 MYC responsive probes in one, but not both experimental designs. Data from the time course and steady state experiments for the non-overlapping regions of the Venn diagram are displayed in Java Treeview format. Region A refers to probes that were called MYC-ER responsive, but not MYC responsive. Region B refers to probes that were called MYC responsive, but not MYC-ER responsive.
(0.46 MB TIF)

Figure S3 c-MYC protein in normal and basal breast carcinoma tissue Paraffin sections of normal breast tissue (A) and basal breast carcinoma tissue (B) were stained with a c-MYC antibody.
(4.32 MB TIF)

Table S1 Unigene IDs of genes identified as responsive to MYCNER and steady state MYCN.
(0.50 MB XLS)

Table S2 GO term enrichment analyses.
(1.61 MB XLS)

Table S3 Comparison on MYC responsive gene identified in this study to the MYC target gene database.
(0.26 MB XLS)

Table S4 Comparison of genes identified in this study and in the Core Serum Response study.
(5.41 MB XLS)

Table S5 Comparison of this study with other studies identifying direct MYC targets.
(0.45 MB XLS)

Table S6 Direction of MYC signature genes that have evidence for MYC binding.
(0.09 MB XLS)
