# Supplementary figures and images for: A Core MYC Gene Expression Signature Is Prominent in Basal-Like Breast Cancer but Only Partially Overlaps the Core Serum Response
Source: PLoS One. 2009 Aug 19;4(8):e6693. doi: 10.1371/journal.pone.0006693 (PMC2723908; doi:10.1371/journal.pone.0006693)

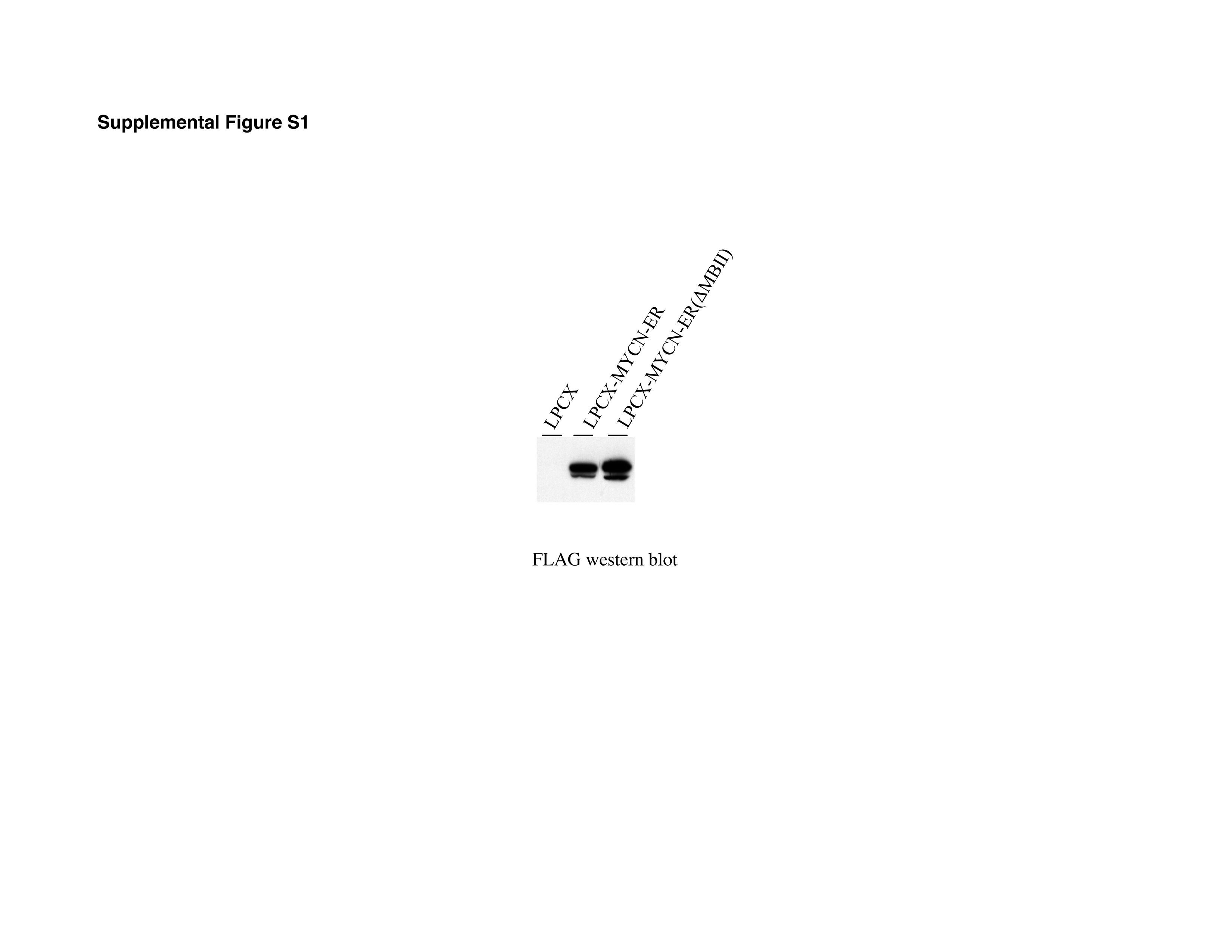

Supplement: Figure S1 — Analysis of expression from transgenes Whole cell lysates of primary human fibroblast cells infected with pLPCX (lane 1) pLPC-MYCN-ER (lane 2), and pLPC-MYCN(del-MBII)-ER (lane 3) were resolved by PAGE and transferred to PVDF for western blot analysis. Because exogenous proteins were FLAG-tagged, western analysis was performed using antibodies specific to the FLAG epitope. (0.19 MB TIF) [file pone.0006693.s001.tif]

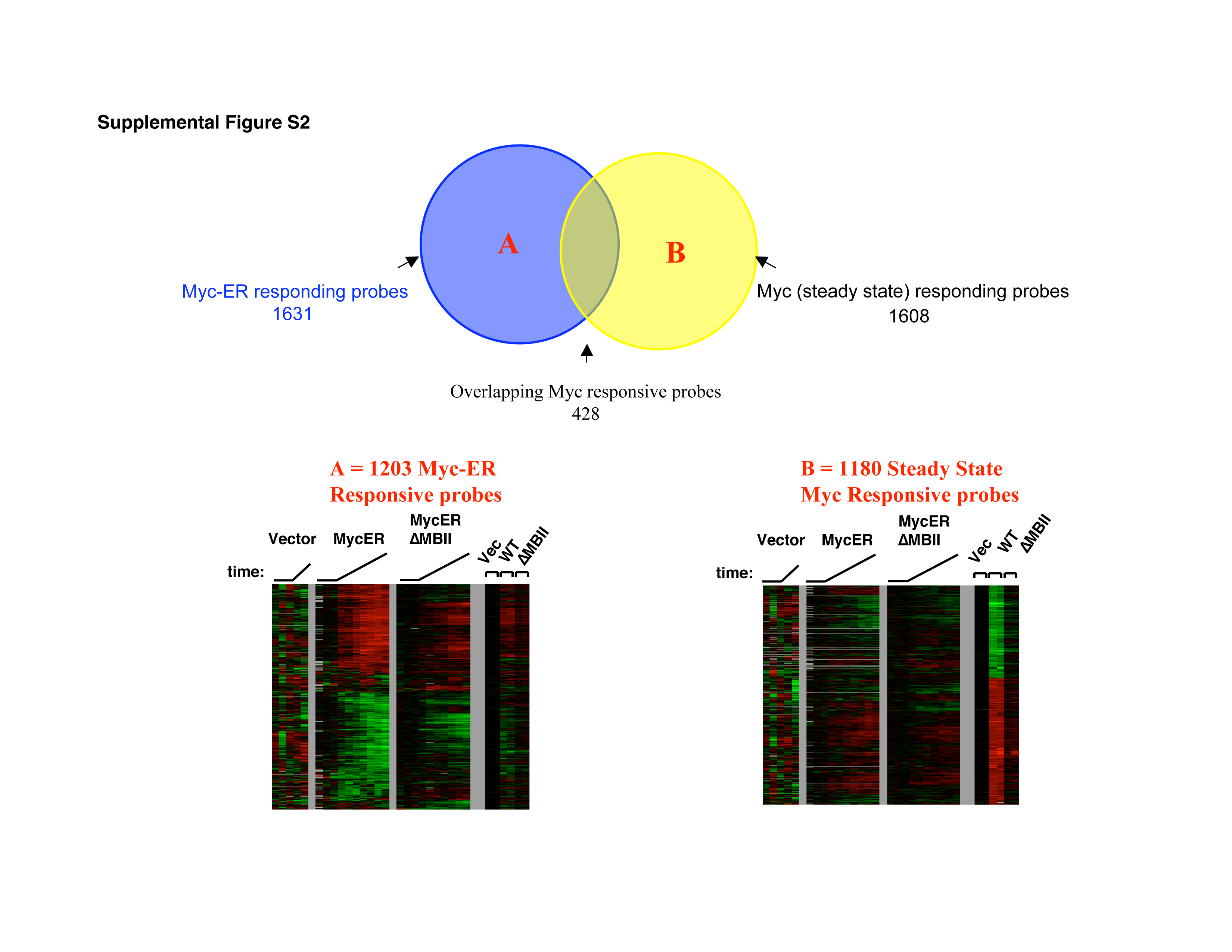

Supplement: Figure S2 — MYC responsive probes in one, but not both experimental designs. Data from the time course and steady state experiments for the non-overlapping regions of the Venn diagram are displayed in Java Treeview format. Region A refers to probes that were called MYC-ER responsive, but not MYC responsive. Region B refers to probes that were called MYC responsive, but not MYC-ER responsive. (0.46 MB TIF) [file pone.0006693.s002.tif]

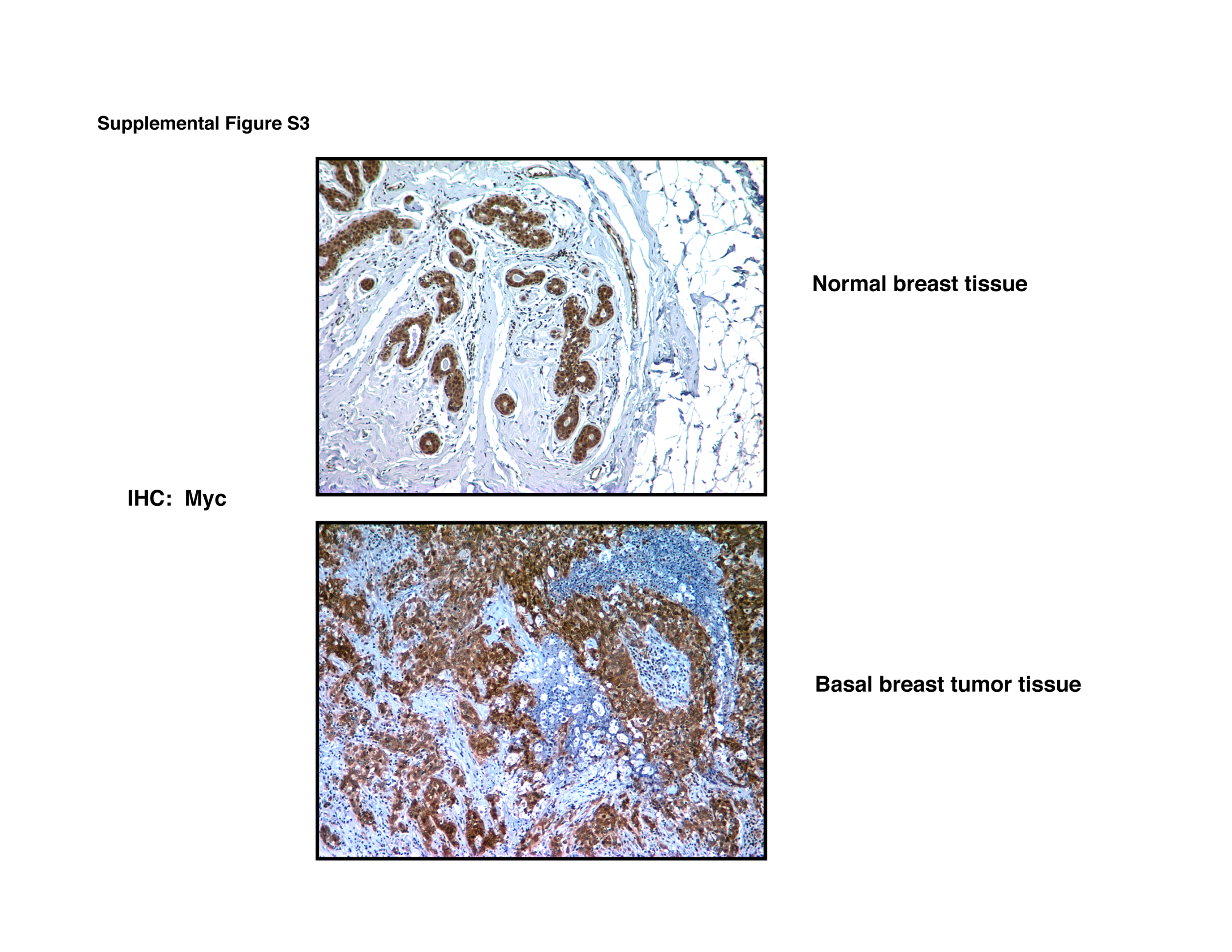

Supplement: Figure S3 — c-MYC protein in normal and basal breast carcinoma tissue Paraffin sections of normal breast tissue (A) and basal breast carcinoma tissue (B) were stained with a c-MYC antibody. (4.32 MB TIF) [file pone.0006693.s003.tif]
